# E167K polymorphism of TM6SF2 gene affects cell cycle of hepatocellular carcinoma cell HEPA 1-6

**DOI:** 10.1186/s12944-017-0468-8

**Published:** 2017-04-13

**Authors:** Shuixian Du, Linlin Lu, Yingxia Miao, Wenwen Jin, Changfei Li, Yongning Xin, Shiying Xuan

**Affiliations:** 1 Medical College of Qingdao University, Qingdao, 266071 China; 2 Department of Gastroenterology, Qingdao Municipal Hospital, 1 Jiaozhou Road, Qingdao, Shandong Province 266011 China; 3 Digestive Disease Key Laboratory of Qingdao, Qingdao, 266071 China

**Keywords:** Hepatocellular carcinoma cell (HCC), Cell cycle, TM6SF2 E167K polymorphism, Western blot, Pcr

## Abstract

**Background:**

Some studties reported that the polymorphism of TM6SF2 gene E167K affects the occurrence and the progression of hepatocytes carcinoma (hepatocellular, HCC). In oeder to investigate the effects of the polymorphism of TM6SF2 gene E167K in the pathogenesis of HCC, we explored its influence on the cell cycle in hepatocellular carcinoma cell HEPA1-6.

**Methods:**

HEPA 1–6 cells which could respectively overexpress TM6SF2 wild type and E167K variant were cultured and HEPA 1–6 cells with zero load plasmids were used as matched control. Flow cytometry was used to detect the cell cycles of these 3 type of HEPA 1–6 cells. Realtime fluores-cence quantitative PCR and western blot were used to analyzed the expression of regulatory factors (Cyclin D1、p53、P16、P27、P21 and Rb) of cell cycle. *T*-test was used in statistical analysis.

**Results:**

Cell cycle phase distribution was presented by the proportion of cells in each phases (%). Compared with the control group, the cell cycle phase distribution (G_1_ phase 57.36 ± 0.21%, G_2_/M phase 25.61 ± 0.36%,S phases 19.31 ± 0.25%) had no differences in wild type group (G_1_ phase 57.63 ± 0.28%, G_2_/M phase 25.77 ± 0.51%, S phases 19.54 ± 0.25%; *P* < 0.05). Between variant type group and wild type group,G_1_ phase was significantly decreased (variant type group G_1_ phase 36.26 ± 0.31%, *P* < 0.05),S phase and G_2_/M phase were increased(variant type group S phase 28.41 ± 0.31%, *P* < 0.05;G_2_/M phase 35.23 ± 0.14%, *P* < 0.05), respectively. Compared with control group,the relative expression of CyclinD1、P53 and Rb mRNA in variant type group was significantly upregulated (2.03 ± 0.01 VS 1.04 ± 0.06, 1.88 ± 0.05 VS 1.37 ± 0.03, 1.29 ± 0.06 VS 1.15 ± 0.03, *P* < 0.05) and P27 mRNA in variant type group was significantly downregulated (0.56 ± 0.02 VS 0.85 ± 0.05, *P* < 0.05).

Compared with wild type group, the relative expression of CyclinD1、P53 and Rb mRNA in variant type group was significantly upregulated (wild type group 1.00 ± 0.00, 1.48 ± 0.09, 1.18 ± 0.01, *P* < 0.05) and P27 mRNA in variant type group was significantly downregulated (variant type group 0.82 ± 0.05,*P* < 0.05). There was no statistical significance between wild type group and control group (*P* > 0.05). P16 and P21 expression showed no statistical sigtfificance in any of these three groups (*P* > 0.05).

**Conclusion:**

E167K polymorphism of TM6SF2 gene affects cell cycles of HEPA1–6 cells via up-regulating CyclinD1、P53 and Rb and down-regulating P27.

## Background

The Strictly cell cycle is the guarantee of proliferation and division, which is very important for maintaining the normal function of the organism. A key function of the cell cycle is to ensure accurate replication and segregation of the genome, because errors in genetic transmission can cause mutations and chromosomal rearrangements that may lead to cell death or disease [1]. Many studies have reported that deregulated cell proliferation and suppressed cell death together provide the underlying platform for neoplastic progression [2–4]. Exploring the changes of cell cycle, which has the great significance to reveal the pathogenesis of the disease. Many studies have reported that the transmembrane 6 superfamily member 2 (TM6SF2) is one of the most important genes of lipid metabolism in the liver and the E167K polymorphism can independently lead to liver fat accumulation, which has an important role in chronic liver disease [5–7]. While some studies reported that the polymorphism of E167K affects the occurrence and the progression of hepatocytes Carcinoma (hepatocellular, HCC) [5, 8, 9]. In order to investigate the effects of the polymorphism of TM6SF2 gene E167K in then pathogenesis of HCC, we explored its influence on the cell cycle in hepatocellular carcinoma cell HEPA 1–6.

In our study, HEPA 1–6 cells which could respectively overexpress TM6SF2 wild type and E167K variant were cultured and HEPA 1–6 cells with zero load plasmids were used as matched control. To explore the cell cycle of HEPA 1–6 cells affected by E167K polymorphism of TM6SF2 gene and the possible mechanisms.

## Methods

### Culture of HEPA 1–6 cell lines

The HEPA 1–6 cell lines were cultured in Dulbecco’s Modified Eagle’s Medium (DMEM) medium containing 100 U/mL penicillin, 10% fetal bovine serum (FBS; Hyclone, USA) and 100 μg/mL streptomycin (Gibco®, USA), and incubated at temperature 37 °C in a humidified atmosphere with 5% CO2. The cells were treated at the time of approximately 80% confluence.

### Construction of Lentiviral vectors

The present study had three groups, including TM6SF2 wild type, TM6SF2 variant type and zero load plasmids as matched control. The lentiviral plasmids (Shanghai Genechem Co., LTD) transfected into 293 T cells, after 24 h culture in DMEM with 10% FBS. 293 T cells were incubated with transfection complexes (expression plasmid, packaging plasmid and transfection reagent) for 48–72 h. We then concentrated the lentivirus and transfected it into HEPA 1–6 cells. The success of the transfection process was validated by performing western blot, real time-polymerase chain reaction (RT-PCR) as well as the percentage of HEPA 1–6 cells with green fluorescent protein (GFP).

### Biochemical indicator assay

Cyclin D1、p53、P16、P27、P21 and Rb monoclonal antibody were purchased from the Biotechnology Corporation, USA. The primer of Cyclin D1、p53、P16、P27、P21 and Rb were designed by Invitrogen Corporation, USA. While the primer of β-actin was synthesized by Nanjing Kingsy Biotechnology Co., ltd.

### Cell cycle determination

The logarithmic growth phase of cells were digested into suspension in a centrifuge tube of 15 ml, centrifugaled at room temperature for 5 min with 800 r/min, centrifugaled and washed 2 times repeatly with PBS, and then fixed at temperature 4 °C with 2 ml 70% absolute alcoholo vernight.

The fixed cells were centrifugaled at room temperature for 5 min with 800 r/min and then retained 0.5 ml PBS to percuss the cells into suspension, and then add 1 mg/ml PI and RNase A, and water bath at temperature 37 °C for 30 min. Flow cytometry was used to detect the cell cycle.

### Western blotting

Total protein of the HEPA 1–6 cells was extracted by RIPA buffer (Sigma-Aldrich, USA). Bradford method was performed to determine the protein concentration following the manufacturer’s protocols, and proteins were frozen at −70 °C until analysis. Antibodies against Cyclin D1(JIANGSU KEYGEN BIOTECH. CO., LTD, KG22272)、p53(JIANGSU KEYGEN BIOTECH. CO., LTD, KG22606)、P16(JIANGSU KEYGEN BIOTECH. CO., LTD, KG22602)、P27(JIANGSU KEYGEN BIOTECH. CO., LTD, KG30242)、P21(JIANGSU KEYGEN BIOTECH. CO., LTD, KG30240)、Rb(JIANGSU KEYGEN BIOTECH. CO., LTD, KG21109) and GAPDH (KGAA002) were used. The expression level of Cyclin D1 or the other protein was normalized relative to the corresponding GAPDH (endogenous reference) level in each lane. Images of Western Blotting were analyzed using Gel-Pro Analyzer Version 4.5 Software (Media Cybernetics, USA).

### Quantitative real time PCR

Total RNA was isolated from the HEPA 1–6 cells using Trizol reagent (Invitrogen: 15,596–026, USA) following the manufacturer’s protocols. Complementary DNA (cDNA) synthesis was performed using the RevertAid First Strand cDNA Synthesis Kit (Thermo Fisher: K1622, USA).

The primers for PCR amplification of the fragments containing Cyclin D1、p53、P16、P27、P21 and Rb were synthesized by Nanjing Sipu Kim Technology Co. Ltd.: 5- CAGATCATCCGCAAACACGC -3 and 5- AAGTTGTTGGGGCTCCTCAG -3 for Cyclin D1, 5-CTGGATTGGCAGCCAGACT-3 and 5- GCTCGACGCTAGGATCTGAC-3 for P53, 5- TGTGCCACACATCTTTGACCT-3 and 5- AGGACCTTCGGTGACTGATGA-3 for P16, 5- CCCTGAACGGAGCTGAAGTC-3 and 5- TAACCGCGCAGCAGATAGTC-3 for P27, 5- CGTTCACAGGTGTTTCTGCG-3 and 5- CATTAGCGCATCACAGTCGC-3 for P21, 5- TGCAGTATGCTTCCACCAGG-3 and 5- TGTTGGTGTTGGCAGACCTT-3 for Rb, 5-CTCCATCCTGGCCTCGCTGT-3 and 5-GCTGTCACCTTCACCGTTCC-3 for β-actin. The PCR amplification profile was as follows: pre-denaturation at 95 °C for 10 min, 35 cycles, denaturation at 95 °C for 15 s, annealing at 58 °C for 60 s, extending at 72 °C for 30 s, and finally extending at 72 °C for 10 min to terminate the reaction. We used the comparative threshold cycle (CT) method to obtain the above genes [10].

### Statistical analysis

Data are expressed as the mean ± standard deviation (S.D.) and T-test was used in statistical analysis. These statistical analyses were performed using SPSS 17.0 statistical software (SPSS Inc., Chicago, IL, USA). *P* < 0.05 were considered statistically significant.

## Results

### Constructed HEPA 1–6 cell lines with overexpress TM6SF2 successfully

The lentiviral plasmids transfected into HEPA 1–6 after 48 h, the positive rate of GFP >95% was observed, which prompted we have constructed stable strains successfully.

### Detected cell cycle by flow cytometry

Table 1 showed the proportion of cells in each cell cycle stage of the three groups. Compared with the wild type group, the cell cycle phase distribution (G_1_,S and G_2_/M) had no differences in control group (t was 1/336, 1.127, 0.444, respectively, *P* all >0.05.); Compared with the wild type group and the control group, G_1_ phase was significantly decreased and S phase, G_2_/M phase were increased in variant type group (*p* all < 0.001.).

**Table 1:** Comparison of the percentage of cell cycle in three groups (*n* = 3)

groups	Cell cycle stage (%)		
	G_1_	S	G_2_/M
variant type group	36.26 ± 0.31	28.41 ± 0.31	35.23 ± 0.14
wild type group	57.63 ± 0.28	19.54 ± 0.25	25.77 ± 0.51
the control group	57.36 ± 0.21	19.31 ± 0.25	25.61 ± 0.36
t1	−88.607	38.577	30.982
p1	<0.001	<0.001	<0.001
t2	−97.595	39.577	29.201
p2	<0.001	<0.001	<0.001
t3	1.336	1.127	0.444
p3	>0.05	>0.05	>0.05

t1:Between variant type group and wild type group; t2: Between variant type group and the control group; t3: Between wild type group and the control group;

### Detection of Cyclin D1、p53、P16、P27、P21 and Rb protein expression by western blot

**Fig. 1:**
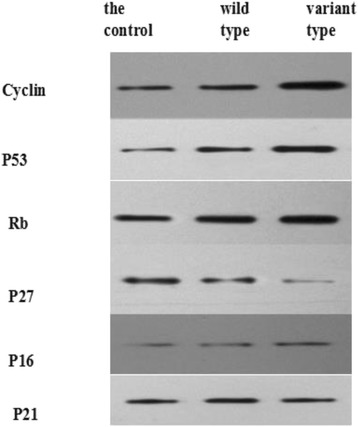
Detection of Cyclin D1、p53、P16、P27、P21 and Rb protein expression by Western blot

Compared with control group and wild type group,the relative expression of CyclinD1、P53 and Rb in variant type group was significantly upregulated and P27 mRNA in variant type group was significantly downregulated (*P* < 0.05), there was no statistical significance between wild type group and control group (*P* > 0.05). P16 and P21 expression showed no statistical sigtfificance in any of these three groups (*P* > 0.05). (Fig. 1).

### Detection of Cyclin D1、p53、P16、P27、P21 and Rb mRNA expression by quantitative real time PCR

Compared with control group, the relative expression of CyclinD1、P53 and Rb mRNA in variant type group was significantly upregulated (t was 28.190, 15.152, 3.615, *P* all <0.05) and P27 mRNA in variant type group was significantly downregulated (t was 9.328, *P* < 0.05). Compared with wild type group, the relative expression of CyclinD1、P53 and Rb mRNA in variant type group was significantly upregulated (t was 178.532, 6.729, 3.132, P all <0.05) and P27 mRNA in variant type group was significantly downregulated (t was 8.363, *P* < 0.05). There was no statistical significance between wild type group and control group (*P* > 0.05). P16 and P21 expression showed no statistical sigtfificance in any of these three groups (*P* > 0.05). (Table 2).

**Table 2:** Detection of Cyclin D1、p53、P16、P27、P21 and Rb mRNA expression by Quantitative Real Time PCR

groups	Quantitative Real Time PCR					
	Cyclin D1	p53	Rb	P27	P21	P16
variant type group	2.03 ± 0.01	1.88 ± 0.05	1.29 ± 0.06	0.56 ± 0.02	0.78 ± 0.06	1.00 ± 0.04
wild type group	1.00 ± 0.00	1.48 ± 0.09	1.18 ± 0.01	0.82 ± 0.05	0.77 ± 0.02	1.01 ± 0.06
the control group	1.04 ± 0.06	1.37 ± 0.03	1.15 ± 0.03	0.85 ± 0.05	0.78 ± 0.04	1.00 ± 0.06
t1	178.532	6.729	3.132	8.363	0.158	0.240
P1	<0.001	0.0016	0.041	<0.001	>0.05	>0.05
t2	28.190	15.152	3.615	9.328	0	0
P2	<0.001	<0.001	0.014	<0.001	>0.05	>0.05
t3	1.156	2.008	1.643	0.735	0.387	0.204
P3	>0.05	>0.05	>0.05	>0.05	>0.05	>0.05

t1:between variant type group and wild type group; t2:between variant type group and the control group; t3:between wild type group and the control group;

## Discussion

Recent genome-wide association studies have identified that variant in TM6SF2 was significantly associated with liver dieases in multiple ethnic groups. Some studies assessed the interaction between TM6SF2 E167K variant in the conditioning of HCC development had come to the conclusion that TM6SF2 E167K variant may be potential genetic risk factors for developing HCC [11–13]. Our study was to explore the cell cycle of HEPA 1–6 cells affected by E167K polymorphism of TM6SF2 gene and the possible mechanisms.

Eukaryotic DNA replication is regulated to ensure all chromosomes replicate once and only once per cell cycle [14]. Errors that result in underreplication or overeplication of the genome in any cell cycle have disastrous consequences and can produce a large array of human genetic diseases, including cancer, birth defects, and many developmental abnormalities [15]. Cell cycle included G_1_, S, G_2_ and M phases. G_1_/S phase and G_2_/M phase were the important “Check” for cell cycle and were to maintain the normal operation of the cell cycle. Cell cycle regulation by protein phosphorylation ensures that pre-RC assembly can only occur in G_1_ phase, whereas helicase activation and loading can only occur in S phase [14]. Once the cell passed through the G_1_/S phase, it will no longer depend on the exogenous proliferation and division signal and complete cell cycle independently [14]. Therefore, the G_1_/S phase is the most critical period of cell cycle regulation. In our study, G_1_ phase was significantly decreased and S phase and G_2_/M phase were increased in variant type group than wild type group and the control group. The result suggested that TM6SF2 gene mutation of E167K may promote DNA replication and accelerate cell cycle of human hepatocellular carcinoma cell line HEPA 1–6 and thereby promote the development of liver cancer cells. Therefore, the acceleration of cell cycle may has the important influence in malignant progression of HCC cells.

Cell cycle regulation is a hot topic in the field of oncology and it is a very complex and delicate process. A variety of internal and external factors involved in the process of cell cycle regulation. These factors included cells cyclin (cyclin), cyclin dependent-kinase (CDK) and some tumor suppressor genes such as Rb, p16, p21, p27, p53 protein products [16, 17]. A large number of studies have showed that cyclin, Rb, p16, p21, p27 and p53 played an important role in G_1_/S phase of cell cycle [18–22]. Uncontrolled cell proliferation is the hallmark of cancer, and tumor cells have typically acquired damage to genes that directly regulate their cell cycles [23]. In our study, our results showed that CyclinD1、P53 and Rb were increased and P27 was decreased in variant type group, which suggested that E167K polymorphism of TM6SF2 gene may change the cell cycle of hepatocellular carcinoma cell HEPA 1–6 through up-regulatating CyclinD1、P53 and Rb and down-regulatating P27.

## Conclusions

In conclusion, this study elucidated that E167K polymorphism of TM6SF2 gene can affect cell cycles of HEPA1–6 cells and the possible mechanisms may up-regulate CyclinD1、P53 and Rb and down-regulate P27. Cell cycle disorder further promoted the deterioration of cell energy metabolism, which thereby formed the vicious cycle to promote progression of HCC.

## Abbreviations

CDK: Cyclin dependent-kinase
Cyclin: Cells cyclin
HCC: Hepatocellular carcinoma cell
PCR: Polymerase chain reaction
RT-PCR: Real time-polymerase chain reaction
TM6SF2: The transmembrane 6 superfamily member 2
